# A neurocomputational analysis of visual bias on bimanual tactile spatial perception during a crossmodal exposure

**DOI:** 10.3389/fncir.2022.933455

**Published:** 2022-11-10

**Authors:** Cristiano Cuppini, Elisa Magosso, Melissa Monti, Mauro Ursino, Jeffrey M. Yau

**Affiliations:** ^1^Department of Electrical, Electronic, and Information Engineering “Guglielmo Marconi,” University of Bologna, Bologna, Italy; ^2^Department of Neuroscience, Baylor College of Medicine, Houston, TX, United States

**Keywords:** neural network, visual bias, bimanual interaction, tactile perception, crossmodal competition

## Abstract

Vision and touch both support spatial information processing. These sensory systems also exhibit highly specific interactions in spatial perception, which may reflect multisensory representations that are learned through visuo-tactile (VT) experiences. Recently, Wani and colleagues reported that task-irrelevant visual cues bias tactile perception, in a brightness-dependent manner, on a task requiring participants to detect unimanual and bimanual cues. Importantly, tactile performance remained spatially biased after VT exposure, even when no visual cues were presented. These effects on bimanual touch conceivably reflect cross-modal learning, but the neural substrates that are changed by VT experience are unclear. We previously described a neural network capable of simulating VT spatial interactions. Here, we exploited this model to test different hypotheses regarding potential network-level changes that may underlie the VT learning effects. Simulation results indicated that VT learning effects are inconsistent with plasticity restricted to unisensory visual and tactile hand representations. Similarly, VT learning effects were also inconsistent with changes restricted to the strength of inter-hemispheric inhibitory interactions. Instead, we found that both the hand representations and the inter-hemispheric inhibitory interactions need to be plastic to fully recapitulate VT learning effects. Our results imply that crossmodal learning of bimanual spatial perception involves multiple changes distributed over a VT processing cortical network.

## Introduction

Multisensory interactions take place at different levels of sensory cortical processing (Giard and Peronnet, 1999; Molholm et al., 2002; Beauchamp et al., 2004; Foxe and Schroeder, 2005; Saint-Amour et al., 2007; Driver and Noesselt, 2008; Stein and Stanford, 2008; Alais et al., 2010; Shams and Kim, 2010; Mercier et al., 2013, 2015; Matchin et al., 2014, for reviews) by different neural structures. Our daily experiences comprise multisensory cues that create and strengthen specific connections among modality-specific sensory regions and weaken others, and these multisensory interactions can shape neural circuits during a protracted period of life. For instance, behavioral benefits in case of audio–visual interactions follow a clear developmental trajectory that extends well into the adolescence (Ross et al., 2007, 2011; Lewkowicz and Ghazanfar, 2009; Brandwein et al., 2011; Burr and Gori, 2012), and in some cases even in the adulthood (Maravita et al., 2002; Holmes et al., 2004). Similarly, extensive experience with visual and tactile stimuli leads to a remapping or reorganization of multisensory representations in healthy subjects and neglect patients (Berti and Frassinetti, 2000; Maravita et al., 2002; Holmes et al., 2004). Generally, the congruence of the multisensory cues can dictate whether the sensory experience results in facilitatory or inhibitory changes (Meredith et al., 1987; Stein and Meredith, 1993; Meredith, 2002; Spence and Squire, 2003; Molholm et al., 2004; Wallace et al., 2004; Navarra et al., 2005; Romei et al., 2007; Rowland and Stein, 2007; Rowland et al., 2007; Van Wassenhove et al., 2007; Musacchia and Schroeder, 2009; Parise et al., 2013; Stevenson and Wallace, 2013; Miller et al., 2015). The behavioral changes associated with multisensory experience necessarily reflect the plasticity of neural structures and cortical networks. Accordingly, how multisensory experience alters cortical systems and behavior is a fundamental question for understanding development, aging, and rehabilitation.

Different sensory systems interact to the extent that they signal redundant or correlated information. For instance, vision and touch both convey spatial information and interactions between these senses have been associated with the recruitment of overlapping cortical systems and analogous neural coding mechanisms (Maunsell et al., 1991; Amedi et al., 2002; Yau et al., 2009). Visual and somatosensory processing interact to support the representation of peripersonal space and limb ownership (Ladavas et al., 1998; Farnè et al., 2003). In non-human primates, bimodal neurons respond to the occurrence of visual and tactile inputs in overlapping receptive fields (RFs) that are anchored to specific body parts (Fogassi et al., 1996; Duhamel et al., 1998; Rizzolatti et al., 1998). These neural populations conceivably mediate the influence of vision on the detection of touch on a single site on the body (e.g., Pasalar et al., 2010). Additionally, experiments in patients with Right Brain Damage (RBD) indicate visual modulation of tactile perception in the contralesional hemisphere: A visual input close to the ipsilesional hand can affect tactile perception on the contralesional hand. These results suggest that visuo-tactile (VT) interactions are not confined to processing on a single hand but also apply to cortical systems that coordinate sensory processing over the two hands (Sherrick, 1964; Verrillo et al., 1983; Craig and Qian, 1997; Soto-Faraco et al., 2004; Spence et al., 2004; Heed and Azañón, 2014; Tamè et al., 2014; Kuroki et al., 2017; Rahman and Yau, 2019). Although chronic perturbations can reveal the existence of multisensory bimanual cortical networks, how multisensory experiences shape these systems remains unclear.

Recently, Wani et al. (2021) reported that task-irrelevant visual cues exert both online and offline influences on tactile spatial perception over the two hands. Participants performed a 4-alternative forced choice (4AFC) task which required them to report on each trial whether they perceived a peri-threshold cue on their left hand only, right hand only, both hands simultaneously, or no stimulation. Participants initially performed the tactile 4AFC task in the absence of visual cues. Performance during this baseline block was unbiased as responses were balanced for the left and right hand cues. Subsequently, participants performed the 4AFC task in VT blocks comprising non-informative visual cues positioned over the left and right hand, that subjects were instructed to ignore. Despite this instruction, concurrent visual cues biased tactile performance and brighter cues induced larger online biases. Moreover, tactile performance was biased toward the hand associated with the brighter cue even on trials when no visual cues were presented during the VT block. These offline effects on bimanual touch conceivably reflect crossmodal learning: the recent history of VT experiences may have reshaped neural circuits supporting tactile detection or spatial attention processing. Importantly, these bias effects could reflect altered representations of the external (peripersonal) space occupied by the brighter visual cues or body-based spatial representations of the hand associated with the brighter visual cues during the VT block.

Here, we sought to leverage the results from Wani et al. (2021) to establish potential neural mechanisms, which can mediate visual influences on bimanual spatial touch. We describe a neural network model previously used to capture crossmodal properties of peripersonal space representations around the left and right hand and recapitulate left-hand tactile extinction in RBD patients (Magosso et al., 2010a,b). First, based on the mechanisms formalized into the network, we identified which synaptic connections could be affected by the protracted experience of congruent and incongruent VT cues. We then described the alternative hypotheses about how VT experience may induce changes in our model, first on the synaptic arrangement and efficacy, and consequently on the network’s behavior. Finally, we simulated the effects of VT exposure on the network and compared the simulation results, realized under the alternative hypotheses, to the data obtained from subjects in Wani’s experiment.

## Materials and methods

### Wani’s experiment

**FIGURE 1:**
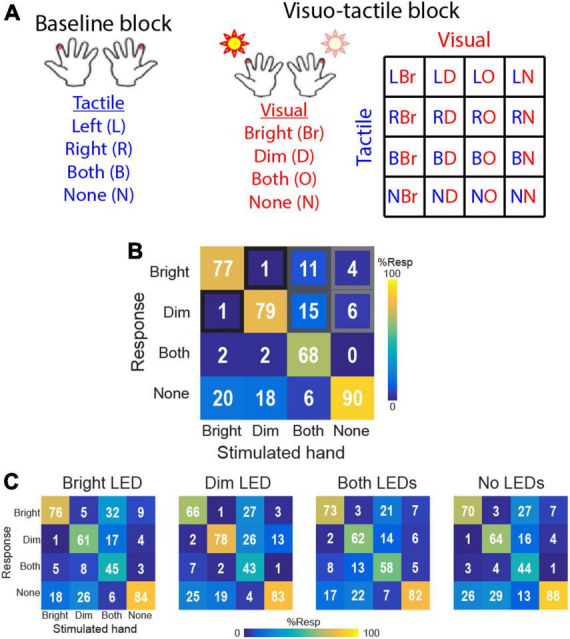
Task and results from Wani et al. (2021). **(A)** 4AFC tactile localization task conditions in baseline block (tactile only) and visuo-tactile block comprising parametric combination of bright and dim LED flashes paired with touch on the left or right hands. Subjects performed the tactile 4AFC task while ignoring the visual cues. **(B)** Baseline block performance. Confusion matrices show group-averaged (*n* = 16) response probabilities on trials comprising tactile stimulation to the hand associated with the bright LED, the hand associated with the dim LED, both hands simultaneously, or no tactile stimulation. **(C)** Visuo-tactile block performance. Group-averaged response probabilities on the 4AFC tactile localization task in the context of bright LED illumination, dim LED illumination, illumination of both LEDs, and no visual distractors.

Here, we briefly summarize the experimental procedure and results described fully in the study by Wani et al. (2021). Subjects were tested using a 4AFC paradigm requiring them to report on each trial whether they detected a brief (5 ms) tactile cue on their left hand, right hand, both hands, or no stimulation. Each hand was associated with either a bright or dim LED (mounted on the tactor; hand associated with the bright LED was counterbalanced across participants). Accordingly, performance data for each hand were analyzed according to whether the hand was associated with the bright or dim LED, even in the baseline block comprising only tactile cues and no visual distractors (Figure 1A). In the absence of visual distractors, participants responded with ∼80% accuracy across conditions (Figure 1B) with highest accuracy on trials comprising no stimulation and lowest accuracy on bimanual trials. Crucially, performance on trials comprising unimanual stimulation on either hand was statistically identical (baseline performance; bright-associated hand: 77 ± 2%, dim-associated hand: 79 ± 2%). Errors, defined as (1) misattributed touch on unimanual trials, (2) unimanual responses on bimanual trials, or (3) false alarms on no-touch trials, were also all equally distributed between the two hands. Thus, there was no evidence for biased tactile detection prior to exposure to VT trials.

Participants then performed the tactile 4AFC task in VT blocks that combined the 4 tactile stimulus conditions to 4 visual distractor conditions: illumination of the bright LED, illumination of the dim LED, illumination of both LEDs or no visual distractors (Figure 1A). These 16 VT conditions were pseudorandomized over the VT blocks (20 reps/condition). With this full factorial design, the visual distractors were uninformative of the tactile conditions.

Data from this experiment showed that the visual stimuli systematically biased tactile detection performance (Figure 1C). When only a single visual distractor was illuminated, participants were more likely to report touch on that side and less likely to report touch on the opposite hand. This pattern suggests that the VT interactions could engage both facilitation and inhibition processes. When both LEDs were illuminated, participants were biased to report touch on the side of the brighter LED. Surprisingly, even on trials with no visual distractors and only tactile cues, participants exhibited biased tactile performance toward the side of the bright LED. Thus, tactile detection performance on the two hands became systematically imbalanced in the VT block, in contrast to the balanced performance in the baseline block.

To quantify visual influences on spatial touch, Wani et al. (2021) introduced the Lateralization Bias Index (LBI) as a metric to quantify the tendency for each participant to report detection on one hand relative to the other hand after accounting for baseline performance:


$$L\,B\,I=B_{c\,o\,r\,r}^{C}-D_{c\,o\,r\,r}^{C};C=V\,i\,s\,u\,a\,l\,B\,r\,i\,g\,h\,t,D\,i\,m,B\,o\,t\,h,N\,o\,n\,e$$

where $B_{corr}^{C}$ and $D_{corr}^{C}$ indicate baseline-corrected response rates for the hand associated with the bright LED (B) and dim LED (D), respectively, for each visual condition (C) separately (i.e., bright LED, dim LED, both LEDs, or no visual distractor). For the bright-associated hand, baseline-corrected rates, $B_{corr}^{C}$, were calculated as the difference in the tactile response rates achieved on trials involving the bright-associated hand in the VT block and the baseline (BL) block:


$$B_{c\,o\,r\,r}^{C}=\left(B_{1\,h\,a\,n\,d}^{C}+B_{2\,h\,a\,n\,d}^{C}+B_{n\,o\,n\,e}^{C}\right)-\left(B_{1\,h\,a\,n\,d}^{B\,L}+B_{2\,h\,a\,n\,d}^{B\,L}+B_{n\,o\,n\,e}^{B\,L}\right)$$

where $B_{1hand}^{x}$ is the hit rate when the bright-associated hand was stimulated alone, $B_{2hand}^{x}$ is the proportion of bimanual stimulation trials when the subjects reported only feeling touch on the bright-associated hand, $B_{none}^{x}$ is the false alarm rate when subjects reported feeling touch on the bright-associated hand when no tactile stimulation was delivered, and *x* indicates the VT block (C) or the baseline block (BL).

Baseline-corrected response rates for the dim-associated hand were similarly computed:


$$D_{c\,o\,r\,r}^{C}=\left(D_{1\,h\,a\,n\,d}^{C}+D_{2\,h\,a\,n\,d}^{C}+D_{n\,o\,n\,e}^{C}\right)-\left(D_{1\,h\,a\,n\,d}^{B\,L}+D_{2\,h\,a\,n\,d}^{B\,L}+D_{n\,o\,n\,e}^{B\,L}\right)$$

The LBI thus described the relative response rates for the bright and dim-associated hands under each VT condition after accounting for potential subject-specific biases in the baseline block. Positive LBI values indicate increased response rates on the hand associated with the bright LED compared to the rates for the hand associated with the dim LED. According to LBI, Wani et al. (2021) found evidence in most individuals for biased responses that differed according to visual conditions.

### General model structure

**FIGURE 2:**
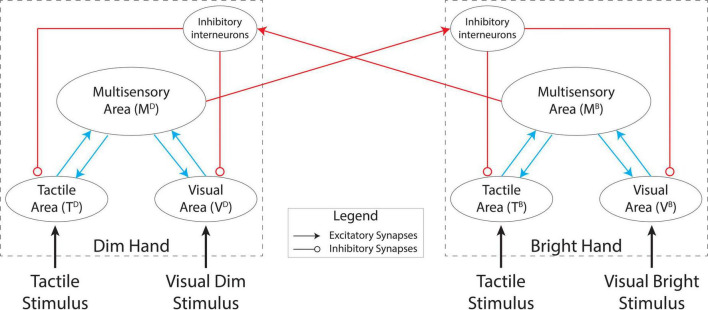
Model architecture. Each hemisphere (dashed boxes) comprises a tactile area, a visual area, and a multisensory area. The unisensory areas reciprocally interact with the multisensory area in each hemisphere. The two hemispheres interact through excitatory projections from the multisensory areas to inhibitory interneurons which modulate activity in the unisensory areas.

In this work, we used a simplified version of the model described previously (Magosso et al., 2010a) which comprised two reciprocally connected, symmetrical networks (see Figure 2) that each simulated a single brain hemisphere. We assumed that each hemisphere supported the perception of tactile stimuli on the contralateral hand of a subject.

In this section, the model is described in a qualitative way; a quantitative description with all equations can be found in Supplementary Appendix.

Mimicking the experimental design by Wani et al. (2021), we assumed that the head and eyes of the simulated subjects are immobile and maintained in central alignment, with each simulated hand located in its own hemispace and in a fixed position. Accordingly, no postural signal is considered and the only inputs received by the model are tactile and visual.

Each network consists of two modality-specific input regions (one visual and one tactile) and a multisensory area which communicates reciprocally with the unisensory regions through excitatory projections. Each hemisphere exerts an inhibitory influence on the other hemisphere by exciting inhibitory units that modulate activity in the unisensory regions.

Units in the two unisensory regions, T and V, are organized in two-dimensional matrices and they respond to tactile and visual stimuli, respectively, applied on one hand. In both regions, each unit has its own receptive field (RF), in hand-centered coordinates, through which it receives stimulation by the external input in the corresponding sensory modality. Units in each unisensory area are topologically organized such that the RFs of the proximal units cover the proximal positions of the hand. Moreover, units belonging to the same unisensory area interact by lateral synapses arranged according to a Mexican hat arrangement (i.e., a circular excitatory region surrounded by a larger inhibitory area) such that neighboring units interact through reciprocal excitation while more distant units interact through reciprocal inhibition.

These unisensory regions exchange feedforward and feedback excitatory connections with a multisensory area that integrates the sensory information from the input areas. The multisensory area could correspond to multisensory regions in the premotor or parietal cortex that receive feedforward projections from sensory-specific areas (Rizzolatti et al., 1981; Graziano et al., 1997; Duhamel et al., 1998). Here, we simulate the multisensory area using a single unit that receives inputs from all of the unisensory units. The feedforward projections from the tactile and visual neurons to the bimodal neuron have the same strength regardless of the position of the unisensory units within their sensory areas. Accordingly, the downstream bimodal unit has a tactile RF covering the entire hand and a visual RF which matches the tactile RF, consistent with the properties of multimodal neurons with RFs spanning the whole hand (Rizzolatti et al., 1981; Graziano et al., 1997). The feedback synapses present the same uniform arrangement as the feedforward synapses, but with different values.

Finally, the multisensory unit is linked through a long-range excitatory projection to an inhibitory unit in the other hemisphere. This “interneuron” inhibits activity in the unisensory units ipsilaterally. The inhibitory projections to the tactile and visual areas have the same arrangement as the feedforward and feedback projections between the unisensory and multisensory units (that is, they have uniform strength, but with a different value).

Based on this synaptic architecture, the multisensory unit in each hemisphere produces effects within and across hemispheres. Within a hemisphere, a multisensory stimulus may reinforce the perception of its unisensory components through the feedback projections to the input areas. This is consistent with neuroimaging studies showing that stimulation in one sensory modality may affect spatial processing in sensory-specific areas of a different modality through back-projections from higher level multimodal areas (Macaluso et al., 2000; Rockland and Ojima, 2003; Macaluso and Driver, 2005). In the case of bimanual stimulation, the multisensory units can also mediate an inter-hemispheric competition through the inhibitory circuit. Indeed, the inhibitory units receive VT information from the multisensory unit in the opposite hemisphere and send inhibitory projections to the unisensory units in the same hemisphere. This competition between the representations of the two hands is consistent with data from RBD patients (Di Pellegrino et al., 1997; Mattingley et al., 1997; Bueti et al., 2004), which implies that the hand representations in the two hemispheres compete for access to limited attentional resources.

All units in the network are normally in a silent state (or low basal activity) and they can be activated if stimulated by a sufficiently large input. The activity of each unit is described through a sigmoidal relationship (with a lower threshold and an upper saturation) and first-order dynamics. A single unit in the model should not be considered as representing a single neuron, but rather as a neuron pool with matching RFs. Similarly, synaptic weights should not be considered as the strength of individual synapses, but rather as an index of overall synaptic efficacy.

### Assignment of model parameters

In basal conditions, the two networks are symmetrical. We utilized the same parameter values used in the previous model (Magosso et al., 2010a), which were implemented to simulate and explain VT interactions like extinction and peri-hand space plasticity, except for the synaptic strengths linking the different model units. Here, synaptic strengths were determined empirically in order for the network to reproduce the baseline performance of the participants in the study by Wani et al. (2021) (Figure 1B). The assignment criteria for network parameter values are reported below. All parameter values are listed in Supplementary Table 1.

#### Receptive field parameters

The RFs of the units in the unisensory regions are implemented as a Gaussian function with unitary amplitude (A) to set a scale for the external inputs and a standard deviation (σ) assigned to obtain RF diameters ranging from 2 to 2.5 cm (Kandel et al., 2000).

#### Parameters of input–output unit response

The static sigmoidal relationship of neural units in the modality-specific regions and multisensory areas is characterized by a smooth transition from inhibition to saturation so that responses progressively decrease as a function of input reduction. This has been used to mimic the bimodal neural response pattern observed when a visual stimulus is progressively moved away from the hand (Colby et al., 1993; Graziano et al., 1997; Duhamel et al., 1998). The sigmoidal function of the inhibitory units has a rapid transition from its resting state to saturation to promote a strong competition between the two hemispheres. The time constant for the first-order dynamics has been assigned to mimic the membrane time constants reported in the literature (Dayan and Abbott, 2001).

#### Synapse parameters

Lateral synapses in the unisensory areas are organized in a Mexican hat arrangement such that an external stimulus can activate only a limited number of unisensory units without the excitation propagating in an uncontrolled manner over the entire population.

The feedforward synapses from the unisensory regions, *Wm*, have a uniform value so that the tactile and visual RFs of the bimodal units cover the entire surface of the corresponding hand (Rizzolatti et al., 1981; Fogassi et al., 1996; Iriki et al., 1996; Graziano et al., 1997; Maravita and Iriki, 2004). This value is set so that the bimodal units are substantially excited by a single unisensory stimulus applied within their RFs (Rizzolatti et al., 1981; Graziano et al., 1997; Duhamel et al., 1998).

The feedback synapses from the bimodal to the visual and tactile input regions, *W*, are uniform with a lower efficacy. This guarantees that a visual stimulus can reinforce the perception of a touch on the same side—and vice versa (Ladavas et al., 1998; Macaluso et al., 2000)—while preventing a unisensory stimulus from producing phantom activations in the other modality-specific area.

The strength of the inter-hemispheric connections (*Wi*) has been chosen so that activation of the bimodal units produces activation of the corresponding inhibitory units to realize a competition between the two hemispheres for attentional resources within the peripersonal space (Mattingley et al., 1997; Dambeck et al., 2006; Battelli et al., 2008). The value for the time constant in the inter-hemispheric connections (Delay; reflecting the time necessary for projections to cross the corpus callosum) has been assigned to agree with *in vivo* data (Fendrich et al., 2004).

The strength of the inhibitory synapses (*I*) has been tuned to allow the simultaneous activation of the two networks in response to bilateral stimulation, even in case of moderate differences in stimuli intensity, in agreement with *in vivo* data in healthy subjects (Hillis et al., 2006).

#### Sensory inputs

The efficacy of the tactile stimulus has been chosen as the minimum value that enables the network to recapitulate the baseline perceptual behavior from the participants in Wani et al. (2021) (see next section for a description of tactile perception by the network). This was done to mimic the experimental procedure performed by Wani et al. (2021) where the amplitudes of the tactile stimuli were determined for each participant’s detection thresholds using an adaptive procedure. To mimic inter-trial variability, we added a noisy component (randomly chosen from a normal distribution with variance 0.2) to modulate this input.

For the visual inputs provided to the model, we set two different strengths (a strong input called “Bright LED” and a weak input called “Dim LED”) defined by a ratio of 2.5-to-1 in accordance with the perceived visual intensities reported by the subjects as described in Wani et al. (2021). Both stimuli were strong enough to produce a correct visual detection in 100% of the presentations. As described for the tactile stimuli, these inputs are also modulated by a noisy component (normal distribution; variance 0.2).

Finally, each hand is labeled according to the visual input associated with it. Hence, we simulate the detection of touch on the hand associated with the bright LED (“bright hand”) and the hand associated with the dim LED (“dim hand”).

Because the aim of this work is to simulate and explain how visual stimuli could bias the tactile perception in the 4AFC task during a protracted VT exposure, we selectively modified some of the parameters related to synaptic connections to test potential mechanisms responsible for the perceptual behaviors found by Wani et al. (2021).

In the following sections we describe the simulations implemented to analyze the visual bias on the tactile perception and how the model behaves in response to the different input configurations utilized to simulate the 4AFC task.

### Network behavior

Perception of a simulated tactile input. As discussed in Magosso et al. (2010b), a critical aspect of modeling perception using neural network models is the assumption of how evoked activity patterns in the network’s regions relate to perception in response to external stimulations. Here, we assumed that a stimulus is perceived if both the specific unisensory input area and the multisensory element exhibit concurrent activity. This agrees with neuroimaging data (Sarri et al., 2006) implying the involvement of both sensory-specific and association cortices in the formation of a conscious percept of an external stimulus. Therefore, we computed a separate “Tactile Awareness Degree” (TAD) for each hand, by taking into account the simultaneous activity evoked in the unisensory and multisensory regions in each hemisphere and comparing their activity with a minimum threshold level (called the “detection threshold,” see Supplementary Appendix for the equations and parameters’ values). Accordingly, if supra-threshold activity occurs in both regions, the model “perceives” the stimulus on that specific hand (TAD > 0, corresponding to the evoked activity of the multisensory element). Conversely, if a sensory input produces only sub-threshold activity in the tactile area, TAD is null resulting in the model failing to perceive the stimulus. On each simulated trial, the activity levels in these regions are evaluated only after the model reaches a steady-state response to the external stimulation.

#### Effect of a tactile stimulus on one hand

A unimanual tactile input (in the absence of visual input) excites the corresponding tactile area which excites the multisensory region in the same hemisphere. This area sends excitatory feedback to the tactile and visual regions associated with the same hand while also exciting the inhibitory unit of the other (unstimulated) hand. In turn, this unit inhibits the visual and tactile regions associated with the unstimulated hand.

#### Effect of a visuo-tactile stimulus on one hand

A unimanual tactile input paired with a spatially congruent visual input excites the corresponding tactile and visual areas which excite strongly the multisensory region in the same hemisphere. This area sends excitatory feedback to the tactile and visual regions associated with the same hand while also exciting the inhibitory unit of the other (unstimulated) hand. In turn, this unit inhibits the visual and tactile regions associated with the unstimulated hand.

Critically, the inhibition generated by the “interneuron” unit is proportional to the excitatory drive of the contralateral multisensory area, so a unisensory stimulus produces a mild inhibition to the other hand, while a multisensory stimulus produces more robust inhibition.

#### Effect of stimuli on both hands

Simultaneous tactile inputs on the two hands excite input areas in both hemispheres and the related multisensory regions. The activated multisensory neuron in one hemisphere excites the inhibitory unit in the other hemisphere which inhibits the unisensory input regions in that hemisphere. This pattern generates a competition between the two hand representations.

### Visuo-tactile exposure effect (simulating the visuo-tactile experience)

Based on the model architecture and the implemented mechanisms, we can expect that the visual bias on tactile localization (Wani et al., 2021) can be mediated by different mechanisms:

1) A modification of the VT representations of each hand, produced by the VT experience, mediated by the excitatory feedforward connections from the unisensory input regions to the multisensory area (blue lines in Figure 2). This modification can happen in two different ways. (A) In the first case, the simultaneous presentation of visual and tactile stimuli on the same hand generates a reinforcement of the feedforward synapses between the input layers and the multisensory region. The overall effect is that a stronger activity in M produces a greater inhibition on the sensory regions of the contralateral hemisphere. (B) The second hypothesis is that, keeping constant the sum of the overall synapses targeting the multisensory element, VT exposure leads to a reorganization of the unisensory inputs to the multisensory region: before the VT experience, the tactile excitatory contribution targeting the multisensory area M is as effective as the visual excitation, because Wm is equal for both modalities. During the multisensory exposure, the visual feedforward connections to the multisensory region are reinforced at the expense of the tactile connections (i.e., fixing the summed synaptic weights necessarily requires reducing the effectiveness of the tactile weights if the visual weights are strengthened). The consequence of this plasticity is that the tactile input alone is less effective in producing a tactile percept. This second hypothesis is consistent with the behavioral data of Wani et al. (2021) that showed that during the VT stimulation, subjects produced fewer reports of tactile stimulation on one hand compared to their detection rates in the Baseline condition. This was true even in case of VT congruent stimulations, where the correct responses were comparable to the baseline tactile behavior. In every other case the tactile accuracy was lower. These patterns support a modification in the synaptic efficacy between the unisensory and multisensory areas.

2) A stronger inhibitory effect of one hand on the other (red lines in Figure 2) that is obtained through the excitation of the interneurons by the multisensory element in the contralateral hemisphere and subsequent inhibition by the interneurons on the ipsilateral unisensory areas. This bias is induced through a Hebbian-like training effect which requires that both hands be simultaneously stimulated and at least one hand receiving a multisensory stimulus. In this way, the multisensory region associated with the hand receiving the VT stimulus is strongly activated, and it excites the related inhibitory interneurons so that both units present an activity above a “training-threshold.” If the opposite hand receives a simultaneous input—unisensory or multisensory—it has at least one input region excited enabling the inhibitory connection with the interneuron to be reinforced. Accordingly, in case of bimanual tactile stimulation, the model presents a bias produced by the stronger inter-hemisphere competition after VT training.

Comparing the three different possibilities, we can argue that in cases 1A and 1B the training does not require that both hands are simultaneously stimulated; it is sufficient for one hand to receive a VT stimulus to reinforce the multisensory representation of this hand in the corresponding hemisphere. Conversely, in interhemispheric inhibition mechanism, the network requires both hands to be simultaneously stimulated with both hemispheres activated and updated in order to produce the observed bias.

To assess the ability of the models to simulate the effect of VT exposure on tactile perception and compare the effects of the alternative hypothetical mechanisms described above, we evaluated the prediction errors of each model by computing the mean squared error (MSE) over all the possible input configurations.

## Results

We first established that the network architecture was able to reproduce the Baseline behavioral results by simulating 16 subjects. We ran several sets of simulations, each one characterized (1) by the same stimuli configurations, with an added noise to mimic stimulus variability among different stimulations, as described in the previous section, and (2) by the same synaptic architecture, with a random variability in its synaptic efficacy, obtained by adding a random component to each connection of the model, chosen from a normal distribution (mean 0 and variance 40% of the synaptic efficacy). The same stimulus configuration was presented 20 times, simulating the number of repetitions of each condition in Wani’s article (Wani et al., 2021), to each of the 16 simulated subjects. We computed the mean results and compared these outcomes to the empirical results described in Wani’s article (Wani et al., 2021). To set the Baseline performance of the network using the Basal parameter values (see Supplementary Table 1), a first set of simulations was performed stimulating the network with just tactile stimuli on one hand (“bright” or “dim”), on both hands simultaneously, or no stimulation.

**FIGURE 3:**
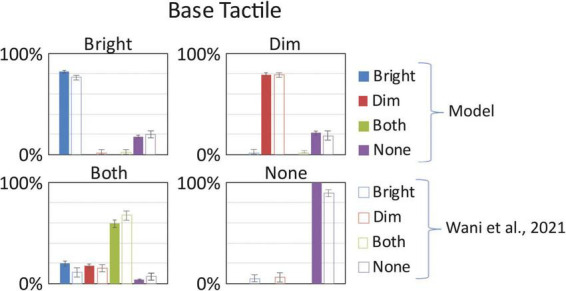
Baseline tactile detection: model vs. (Wani et al., 2021). In each box we reported the percent tactile responses (*y*-axis), distributed across the four possible choices (Bright, Dim, Both, None, on the *x*-axis), mediated over the 20 simulated subjects, to a specific input configuration, specified in each box title: tactile stimulus only on the bright hand, “Bright,” on the dim hand, “Dim,” on both hands simultaneously, “Both,” and no stimulus, “None.” Errorbars indicate s.e.m. The responses obtained by the model, with its basal parameters’ values as in Supplementary Table 1, are compared to the empirical results of Wani et al. (2021).

As shown in Figure 3, the model reproduces quite well the behavioral results. The MSE computed in this Baseline configuration (MSE < 0.2%) confirms a good model performance in simulating the behavioral results. In case of stimulation on a single hand (both “Bright” or “Dim” equally), the model reaches an accuracy of about 80%, a level comparable with the experimental results. Likewise, in case of null tactile stimuli on both hands, the model presents a correct detection in 100% of the repeats. Finally, when both hands are stimulated with a tactile input, the response pattern obtained by the network is distributed across the 4 alternative responses: in 60% of the simulations the network correctly identifies both inputs; in about 3% of the cases it fails to detect any stimulus; while in the rest of the simulations the detection is almost equally distributed across the two simulated hands, 19.7% on the Bright hand and 17.5% on the Dim hand. These results mimic the group-level behavior found by Wani et al. (2021). From this initial analysis we show that network is strongly sensitive to unilateral tactile stimuli, and somewhat error-prone with bilateral stimuli (which produced a unilateral percept). Importantly, as with the human behavior, the model’s performance is balanced between the hands. These preliminary simulations enabled us to identify the basal parameter values and established the network’s baseline behavior prior to VT training.

Wani et al. (2021) found that a protracted experience of VT stimulation led to a tactile bias to the hand associated with the brighter visual cue. Importantly, this bias persisted after VT experience even when subjects experienced tactile stimulation only. To explain this cross-modal influence, we ran a second set of simulations. Our aim was to test different hypotheses regarding the emergence of this visual influence on tactile perception during a VT-repeated exposure.

The first mechanism tested was the effect of plasticity restricted to the feedforward synapses between the unisensory and the multisensory elements for each hand. We tested the results of such effect by increasing the effectiveness of the feedforward synapses between the input regions and the corresponding multisensory element. Because of the asymmetric visual stimulation that subjects received on their hands during the VT tasks (i.e., one visual cue was brighter), we assumed asymmetric reinforcement between the two hands. We tested different asymmetric conditions to analyze the potential effect of such mechanism on the tactile perception. Thus, the “Bright” hand would increase by 50–80% the effectiveness of its feedforward connectivity with the multisensory region, while the “Dim” hand connectivity increased only by 10–30%. Every other parameter of the network was maintained in its baseline configuration (see Supplementary Table 1). The behavior of the network, under the different possible synaptic configurations, was quite consistent.

**FIGURE 4:**
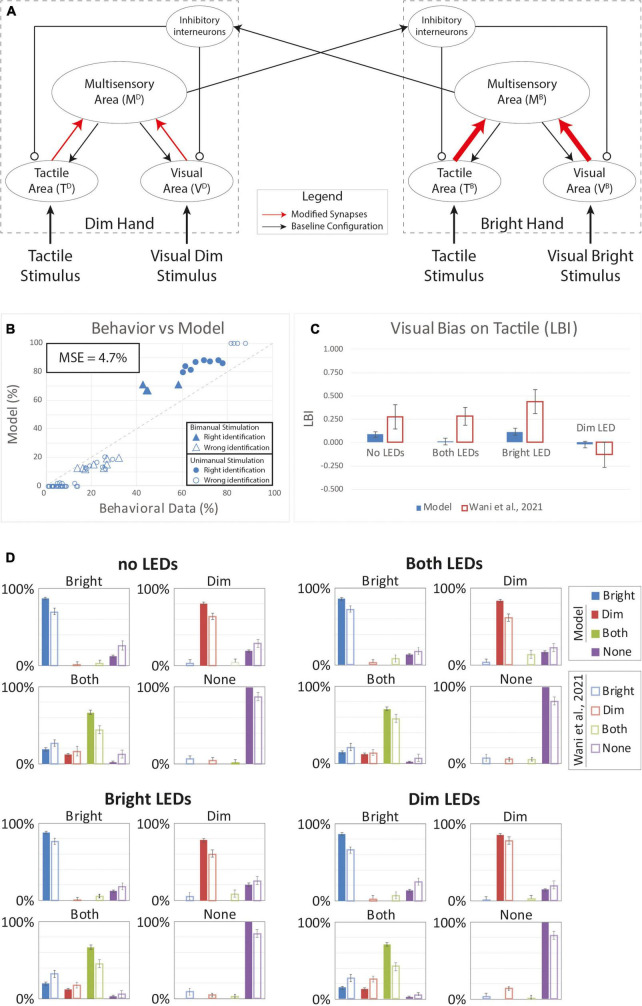
Simulated results of the tactile perception with increased effectiveness of feedforward excitatory synapses. **(A)** Model’s structure with the altered pattern of connectivity. Red arrows highlighted the modified synapses. Thick synapses represent stronger reinforcement; thin arrows correspond to a weaker reinforcement. **(B)** Behavioral data from Wani et al. (2021) are compared with Model’s results, for the 16 different stimuli configurations. The dashed line represents the “perfect” match between simulations and behavioral results. In the diagram, the triangles represent the tactile responses in case of bimanual tactile stimulations, irrespective of the position of the visual stimuli. Full triangles refer to the percent correct identification of the bimanual tactile stimulation; empty triangles report the other responses (T on the bright or on the dim hand). Circles report the responses to unimanual tactile stimulations, regardless of the positions of the visual stimuli. Full circles represent the correct responses, empty circles report the other responses. **(C)** Lateralization Bias Index (LBI) computed for the 4 different visual conditions. **(D)** In each box we reported the percent tactile responses (*y*-axis), distributed across the four possible choices (Bright, Dim, Both, None, on the *x*-axis), mediated over the 20 simulated subjects, to a specific tactile input (T) configuration, specified in each box title: T on the bright hand, “Bright,” on the dim hand, “Dim,” on both hands simultaneously, “Both,” and no stimulus, “None,” and for each visual input stimulation: no LEDs, both LEDs, Bright LED, Dim LED. Errorbars indicate s.e.m. The responses obtained by the model, are compared to the empirical results of (Wani et al., 2021).

Figure 4 shows results for an increase equal to 80% for the “Bright” hand and 10% of the “Dim” hand. Compared to human behavior, the model overperforms in case of correct responses and has a lower rate of “misattributed touch” responses (Figure 4B). The MSE (4.7%) confirms this disparity. Moreover, as shown by the LBI histogram (Figure 4C), there is minimal visual bias on tactile perception and the model bias is lower than the bias in human behavior for all VT configurations. We conclude that this model performs poorly given the LBI results and that the model hit rates are always greater than observed in human behavior (Figure 4D).

We next tested that the hypothesis that VT experience could produce a multisensory reorganization of the sensory representations of the two hands. Behavioral data for tactile detection showed that, during the VT stimulation block, subjects’ accuracy on single hand stimulation never exceeded performance in the Baseline condition (“T only” before the VT experience). Reduced performance in the VT block was evident even when comparing the relatively high hit rates for congruent VT single-hand stimulation (T and V stimuli only on the Bright hand and T and V stimuli only on the Dim hand) to the baseline single-hand performance. This could suggest an adjustment in the synaptic efficacy between the unisensory and multisensory areas within each hemisphere. To assess this possibility, we performed simulations modifying the strength of the projections (while maintaining the overall synaptic weights; see “Methods”) between these regions: The effectiveness of the synapses between the tactile regions and the multisensory area were lowered while the synaptic weights associated with the visual input areas were increased.

**FIGURE 5:**
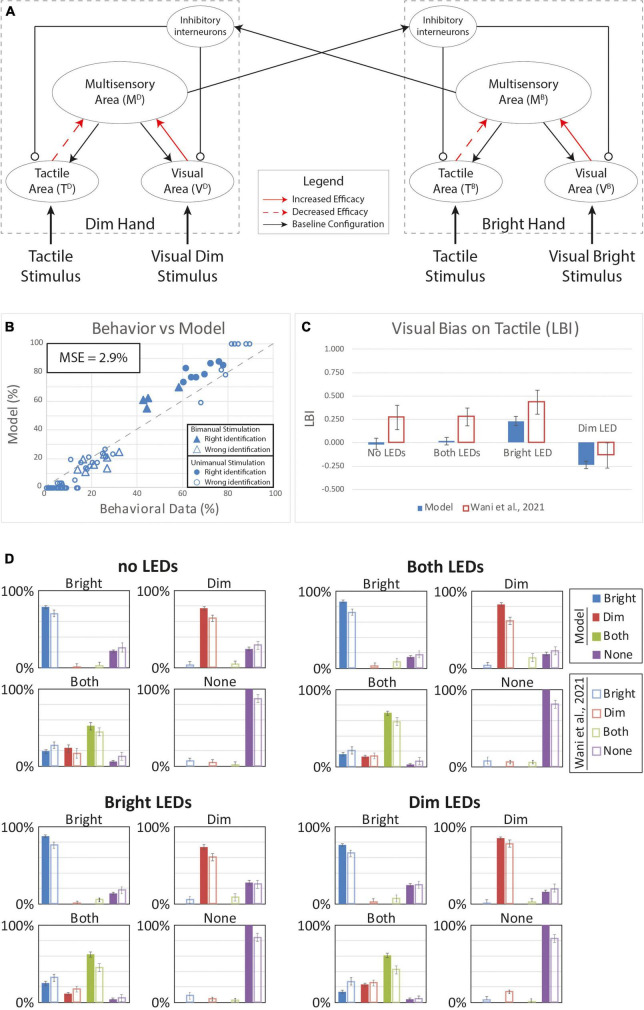
Tactile perception with a reorganization of the synaptic representation of the two hands. Visual connectivity was increased by 20% and the tactile efficacy was lowered of the same quantity. Overall, the sum of the synaptic efficacy targeting the multisensory area and coming from the modality specific input regions was kept constant. **(A)** Model’s structure with the altered pattern of connectivity. Red solid arrows identified synapses with increased efficacy, red dashed arrows the synapses with lower efficacy. Panel **(B)** depict the comparison between model’s responses and experimental data, in case of the different tactile and visual stimulations. In the diagram the triangles represent the tactile responses in case of bimanual tactile stimulations, irrespective of the visual stimuli. Full triangles refer to the correct identification of bimanual stimulations; empty triangles report the percentages of different sensory perceptions. Circles report the responses to unimanual tactile stimulations, regardless of the positions of the visual stimuli. Full circles represent the correct responses, empty circles report the other responses. Panel **(C)** reports the LBI for the different visual configurations. **(D)** In each box we reported the percent tactile responses (*y*-axis), distributed across the four possible choices (Bright, Dim, Both, None, on the *x*-axis), mediated over the 20 simulated subjects, to a specific tactile input (T) configuration, specified in each box title: T on the bright hand, “Bright,” on the dim hand, “Dim,” on both hands simultaneously, “Both,” and no stimulus, “None”; and for each visual input stimulation: no LEDs, both LEDs, Bright LED, Dim LED. Errorbars indicate s.e.m. The responses obtained by the model, are compared to the empirical results of Wani et al. (2021).

Figure 5 shows results when the tactile weights were reduced by 20% and the visual weights were strengthened by 20%. With unimanual tactile stimulation on the bright and dim hands, model hit rates ($p_{B\,r}^{R\,e\,o\,r\,g}=78\%,p_{D\,i\,m}^{R\,e\,o\,r\,g}=77\%$) are slightly lower compared to Baseline model performance ($p_{B\,r}^{B\,a\,s\,e}=82\%,p_{D\,i\,m}^{B\,a\,s\,e}=79\%$). This matches the behavior in the human sample (MSE = 2.9%) generally; however, the model behavior differs from human performance in notable ways. Specifically, while the model captures the LBI changes (Figure 5C) in the “Bright Led” and the “Dim Led” conditions, it fails to reproduce bias in the “No LEDs” and “Both LEDs” conditions. Thus, this model still fails to reproduce the full range of VT bias effects.

We next tested the potential effects of plasticity in the interhemispheric competition between the sen-sory representations of the two hands. Based on our model architecture, if one hand is stimulated by a visual or a tactile input, the corresponding multisensory region can stimulate the interneuron in the opposite hemisphere which sends inhibitory feedback projections to suppress the activity in the corresponding sensory input regions (Figure 2). This inhibition can prevent the detection of touch on the contralateral hand. We assumed that protracted VT experience could strengthen the synaptic architecture implementing such competition. We tested this alternative hypothesis by reinforcing these connections by strengthening the inhibitory effect of the “Bright” hand onto the “Dim” hand. In our model, we reinforced the connections from the multisensory region of the Bright hand to the contralateral interneuron and the inhibitory synapses from this element to the modality specific input regions of the Dim hand by 50%. Additionally, we reinforced the connections from the multisensory region of the Dim hand to the contralateral interneuron and the inhibitory synapses from this element to the modality specific input regions of the Bright hand by only 10%.

**FIGURE 6:**
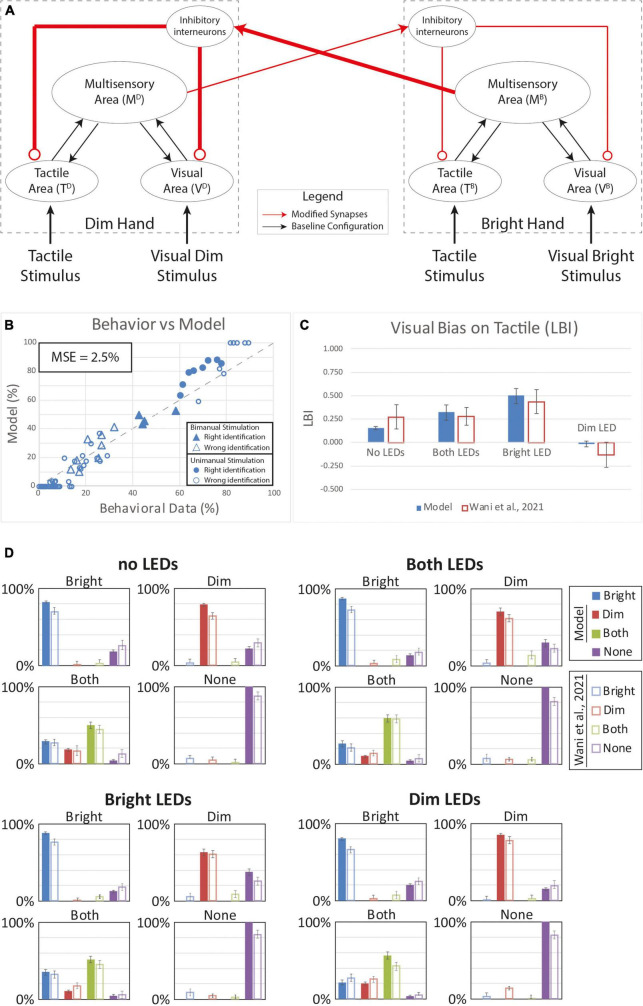
Simulated tactile perception with stronger competition between the two hands’ neural representations. **(A)** Model’s structure with the altered pattern of connectivity. Red lines highlighted the modified synapses. Thick synapses represent stronger reinforcement; thin lines correspond to a weaker reinforcement. **(B)** Behavioral data from Wani et al. (2021) are compared with Model’s results, for the 16 different stimuli configurations. The dashed line represents the “perfect” match between simulations and behavioral results. In the diagram the triangles represent the tactile responses in case of bimanual tactile stimulations, irrespective of the visual stimuli. Full triangles refer to the correct identification of bimanual stimulations; empty triangles report the percentages of different sensory perceptions. Circles report the responses to unimanual tactile stimulations, regardless of the positions of the visual stimuli. Full circles represent the correct responses, empty circles report the other responses. **(C)** Lateralization Bias Index (LBI) computed for the 4 different visual conditions. **(D)** In each box we reported the percent tactile responses (*y*-axis), distributed across the four possible choices (Bright, Dim, Both, None, on the *x*-axis), mediated over the 20 simulated subjects, to a specific tactile input (T) configuration, specified in each box title: T on the bright hand, “Bright,” on the dim hand, “Dim,” on both hands simultaneously, “Both,” and no stimulus, “None”; and for each visual input stimulation: no LEDs, both LEDs, Bright LED, Dim LED. Errorbars indicate s.e.m. The responses obtained by the model, are compared to the empirical results of Wani et al. (2021).

Figure 6 depicts the results with stronger and asymmetric inhibition between the two hands. With this synaptic configuration, the behavior of the model looks more similar (MSE = 2.5%) to the group-level behavioral data relative to the previous hypotheses based on within-hemisphere plasticity (Figures 4, 5). The model reproduces much of the distributed response pattern found by Wani et al. (2021) with bimanual tactile stimulation, with and without concurrent visual stimulation (see triangles in Figure 6B). This model successfully accounts for the direction of all of the bias effects (Figure 6C), though it underestimates the strength of the bias observed with visual stimulation on the “Dim” hand (LBI = -0.02). Thus, our model assuming changes in the strength of interhemispheric interactions performance better compared to the alternative models based on within-hemisphere changes; however, it still failed to account for bias direction and strength in some conditions.

**FIGURE 7:**
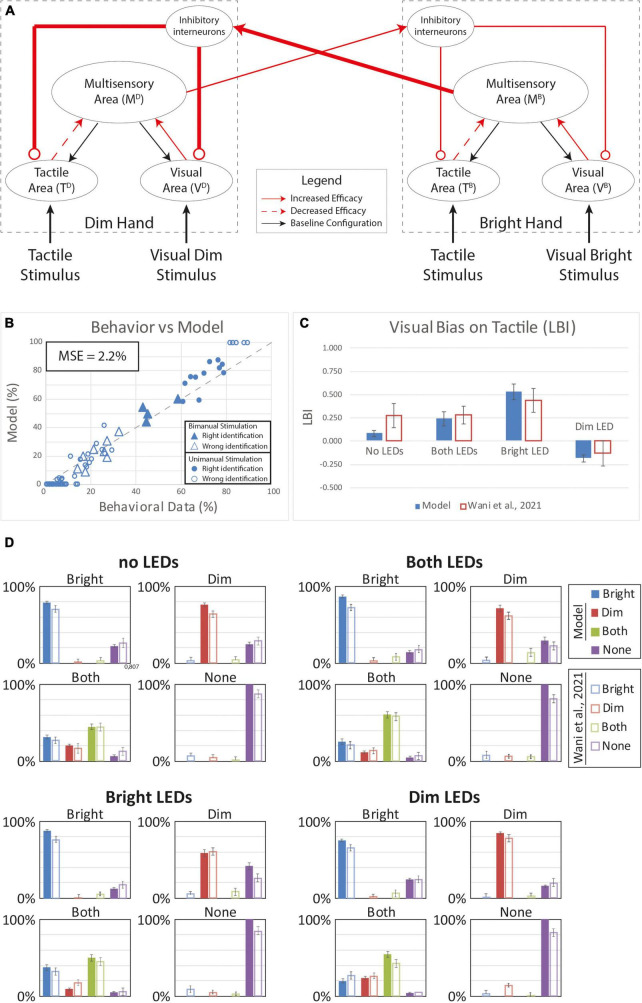
Simulated tactile perception in case of sensory reorganization paired with a stronger inter-hemisphere competition. **(A)** Model’s structure with the altered pattern of connectivity. Red lines highlighted the modified synapses. Thick synapses represent stronger reinforcement; thin lines correspond to a weaker reinforcement. Moreover, for each hand representation, red solid arrows identified synapses with increased efficacy, red dashed arrows the synapses with lower efficacy. **(B)** Behavioral data from Wani et al. (2021) are compared with Model’s results, for the 16 different stimuli configurations. The dashed line represents the “perfect” match between simulations and behavioral results. In the diagram the triangles represent the tactile responses in case of bimanual tactile stimulations, irrespective of the visual stimuli. Full triangles refer to the correct identification of bimanual stimulations; empty triangles report the percentages of different sensory perceptions. Circles report the responses to unimanual tactile stimulations, regardless of the positions of the visual stimuli. Full circles represent the correct responses, empty circles report the other responses. **(C)** Lateralization Bias Index (LBI) computed for the 4 different visual conditions. **(D)** In each box we reported the percent tactile responses (*y*-axis), distributed across the four possible choices (Bright, Dim, Both, None, on the *x*-axis), mediated over the 20 simulated subjects, to a specific tactile input (T) configuration, specified in each box title: T on the bright hand, “Bright,” on the dim hand, “Dim,” on both hands simultaneously, “Both,” and no stimulus, “None”; and for each visual input stimulation: no LEDs, both LEDs, Bright LED, Dim LED. Errorbars indicate s.e.m. The responses obtained by the model, are compared to the empirical results of Wani et al. (2021).

Because the tested models accounted for different aspects of the behavior, we reasoned that a model assuming a combination of plasticity mechanisms could capture for full range of VT bias effects. Accordingly, we tested a final model that assumed plasticity in inter-hemispheric competition as well as multisensory reorganization of each hand representation within the hemispheres (Figure 7). Indeed, this dual-mechanism model better reproduced human performance under the unisensory and VT stimulus conditions (MSE = 2.2%) compared to the alternative single-mechanism models (Figure 7B). Notably, the model predicted substantial bias in each visual conditions (Figures 7C,D) and correctly accounted for the direction of bias. Thus, the model assuming plasticity in the multisensory hand representations within each hemisphere and changes in the competition between the two hand representations recapitulated the online and offline effects of visual experience on bimanual touch.

## Discussion

Wani et al. (2021) analyzed the effect of visual-tactile exposure on the tactile perception of unimanual and bimanual stimulation in a 4AFC task. Although performance during a baseline block comprising no visual cues was balanced for the left and right hand cues, performance became biased during test blocks in which non-informative visual cues were presented over the left and right hands. Moreover, tactile performance was generally reduced and biased toward the hand associated with the brighter cue even on trials when no visual cues were presented after VT exposure. According to signal detection theory models, the visual cues induced the performance reductions and spatial biases through online reductions in criterion (resulting in greater false alarms) and offline brightness-dependent reductions in sensitivity (resulting in lower hit rates). Here, we sought to link these behavioral results to network plasticity in a neurocomputational model (Magosso et al., 2010a,b) previously implemented to investigate VT interactions like left-hand tactile extinction on RBD patients (Di Pellegrino et al., 1997; Ladavas et al., 1998; Ladavas and Farnè, 2004), VT integration in peripersonal space (Fogassi et al., 1996; Graziano et al., 1997; Duhamel et al., 1998; Rizzolatti et al., 1998) and the expansion of spatial RFs through experience with a handheld tool (Magosso et al., 2010a). Our main findings are that the VT effects are inconsistent with just plasticity of unisensory visual and tactile representations of each hand or just plasticity in the strength of inter-hemispheric inhibitory interactions. Instead both the hand representation and the inter-hemispheric inhibitory interactions need to be plastic to capture all the behavioral results.

Our model describes the interaction between the two hands by means of inter-hemispheric inhibition (Figure 2). Assuming that each hemisphere contains visual and somatosensory representations of one hand, activation of one hand representation by sensory inputs exerts an inhibitory effect on the representations of the opposite hand through activation of an inhibitory interneuron population through long range projections from a multisensory area. This network architecture can reproduce bimanual suppressive interactions such as masking (Sherrick, 1964; Gilson, 1969; Verrillo et al., 1983; Tamè et al., 2011) as well as a number of bilateral VT interactions. By tuning a subset of the model parameters, we reproduced the behavioral patterns from the 4AFC bimanual localization task, in the baseline condition (T only stimulations). The modifications of those parameters (i.e., synaptic weights) were needed to accommodate the behavioral variance present in the participant sample tested in Wani et al. (2021).

Our model is a clear oversimplification of the cortical systems that support multisensory and bimanual processing. For instance, while our model assumes inter-hemispheric inhibition of the two hand representations is driven by activity in the multisensory area, callosal connections are known to exist between the hand representations in the area 2 subdivision of primary somatosensory cortex (Killackey et al., 1983) and higher-order somatosensory regions area 5 (Iwamura et al., 2001) and secondary somatosensory cortex (Jones and Powell, 1969; Disbrow et al., 2001). Similarly, our model comprises a single multisensory area in which visual and tactile hand information converges, which nominally represents the ventral intraparietal area (Duhamel et al., 1998), but neurons responsive to visual and tactile stimulation can be found in several parietal association areas and subdivisions of the intraparietal sulcus (Delhaye et al., 2018). Finally, inhibitory connections only exist between the interneuron node and the unisensory populations in our model; however, inhibitory circuits are ubiquitous across sensory areas and suppressive bimanual interactions in primary somatosensory cortex thought to reflect feedback from higher-order somatosensory regions (Burton et al., 1998; Lipton et al., 2006; Tommerdahl et al., 2006; Reed et al., 2011). Accordingly, rather than trying to link our model components to specific neural substrates in the primate brain, we instead present the architecture as a streamlined framework that accounts generally for unisensory responses, multisensory processes, and competitive inhibition between the left and right hand representations.

By manually setting model parameters, we tested different hypotheses for how the network architecture could be modified to recapitulate the behavioral patterns associated with VT experience. These alternative models represent how the central nervous system may adapt to sensory experiences. We identified two network mechanisms (feedforward synapses within each hemisphere and inhibitory inter-hemispheric interaction) as possible mechanisms to reproduce the behavior in Wani et al. (2021). In the first case, the experience of multisensory cues on each single hand induces reorganization of the visual and tactile hand representations while leaving inter-hemispheric interactions unaltered. The within-hemisphere changes are enacted in the unisensory regions through excitatory feedback projections from the multisensory area and in associative cortex through the reinforcement of feedforward projections from the unisensory regions to the multisensory area. We considered two models for altering the strength of the feedforward synapses. In one model, we strengthened all feedforward projections from the visual and somatosensory regions to the multisensory area in a manner that depended on the strength of the visual responses (which depended on the brightness of the visual cues). Accordingly, the hand associated with the bright LED during the VT exposure would experience greater stimulation thereby resulting in a stronger reinforcement of the connections between the unisensory and multisensory areas representing that hand. Conversely, the hand associated with the dim LED would experience weaker stimulation and its neural representation would undergo weaker reinforcement. A second method for modifying the feedforward connection strengths was to rebalance the weights based on visual experience while assuming a fixed total weight summed over all visual and tactile feedforward projections. In this model, the reinforcement of the visual connections to the multisensory area would induce a reduction in the strength of the tactile projections. While the first condition showed poor results, in terms of the ability to simulate and explain the VT effect on the tactile perception, the second method exhibited a better ability to reproduce the experimental data. Nevertheless, even if it can successfully relate some behavioral changes following VT experience to network changes confined to the connections supporting the hand representations within each hemisphere, without modifying the strength of the inter-hemispheric projections, it is still not able to fully account for the full observed behaviors.

An alternative hypothesis was that changes observed following VT experience could be related to changes in the inter-hemispheric interactions. In this model, the excitatory feedforward and feedback projections supporting the hand representations in each hemisphere are unaffected by VT experience. Instead, the model posits that VT experience reinforces the projection weights linking the multisensory area in one hemisphere to the inhibitory interneurons in the opposite hemisphere while also modifying the inhibitory feedback connections targeting the unisensory hand representations. Accordingly, in Wani’s experiment (Wani et al., 2021), the initially balanced competitive interaction between the left and right hand representations becomes biased in favor of the hand associated with the bright LED after VT exposure as the stronger activity in the multisensory region leads to strengthened inhibition of the hand unisensory representations associated with the dim LED. While this model shows results with a better correspondence with the observed behavior in Wani et al. (2021), yet it is not able to fully support their findings.

Since both of the models previously discussed, assuming exclusive changes to the sensory hand representations or the inter-hemispheric interactions, could not reproduce the observed results, we hypothesized that a combination of these mechanisms could be responsible for the overall behavior in Wani et al. (2021). Accordingly, we implemented a model characterized by a rebalanced feedforward connection strengths along with changes in the inter-hemispheric interactions. These combined effects, based on visual experience, showed the best fit for the behavioral results described by Wani et al. (2021).

This model makes distinct predictions in other contexts that remain to be tested. The two mechanisms implemented in the model impose specific sensory requirements for induction of the VT adaptation effects. First, a VT experience on a single hand is required to alter the corresponding hand representation in the network, accordingly to the mechanism assuming changes to the hand representations, and it would reproduce results in case of visual stimulation on a single hand; yet this experience would not be enough to account for the behavior showed in case of bimanual stimulation. Second, the model always requires bimanual sensory experiences in order to induce network changes, under the inter-hemispheric competition mechanism, and to reproduce the behavioral results in case of bimanual VT conditions. Thus, the model predicts that an experiment that restricts VT experience to only unimanual conditions, unlike Wani et al. (2021) which tested both unimanual and bimanual VT cues, would not induce differences in case of VT bimanual stimulation. Third, in case of a combination of VT experience on one hand and a unisensory experience on the opposite hand it would be sufficient to affect the overall competition between the sensory representation of the hands in the two hemispheres, but the competition would be restricted to the sensory modality presented to the second hand.

The current work has clear limitations. As discussed above, we did not intend to link our model to specific neural substrates, so we are poorly positioned to even speculate on specific neurophysiological changes associated with VT experience. That said, based on the hypothesis that hand representations are plastic, we might predict that neural activity levels associated with single hand stimulation, perhaps widely distributed over the somatosensory cortical system (Delhaye et al., 2018), would be enhanced or reduced with VT experience. Based on the hypothesis that the inhibitory interactions are plastic, we might predict that the suppressive interactions observed with bimanual stimulation (Burton et al., 1998; Lipton et al., 2006; Tommerdahl et al., 2006; Reed et al., 2011) would be modulated by VT experience. We hypothesized that the behavioral adaptations following VT experiences resulted from a Hebbian process and we sought to identify potential network-level changes to explain the behavioral effects.

Simulations supported the integration of two mechanisms: modified competition between the two hemispheres is needed to explain the bimanual results, but this must be coupled with a reorganization of the unisensory representations of the two hands to fully capture the unimanual results. This hypothesis must be tested with actual Hebbian training paradigm. The limited data from Wani et al. (2021) was insufficient for further constraining and analyzing the role played by each of our suggested mechanisms. Likely due to this limitation, the current models also struggled to account for the reduction of unimanual performance after VT exposure, compared to baseline. Additional experiments designed to probe Hebbian learning processes which systematically restrict sensory experiences to unimanual or bimanual conditions in the VT exposure phase could conceivably dissociate network-level modifications underlying unimanual performance changes from those underlying bimanual performance changes. This disambiguation may be further aided by exploring the trajectory of modified tactile performance on the 4AFC bimanual task. The current efforts are based on time-averaged behavioral effects computed over the whole VT exposure period. It may be reasonable to suppose that the unimanual and bimanual behaviors following initial exposure to VT cues may differ substantially from behavior after extensive VT experience and these differences may reveal critical insights into the role of perceptual experience in shaping multisensory hand representations and inter-hemispheric competition.

## Data availability statement

The original contributions presented in this study are included in the article/Supplementary material, further inquiries can be directed to the corresponding author.

## Author contributions

CC ran the simulations, analyzed results, and wrote the manuscript. EM implemented the model. MM ran the simulations. MU discussed the results. JY discussed the results and wrote the manuscript. All authors contributed to the article and approved the submitted version.
