# Supplementary material for: A neurocomputational analysis of visual bias on bimanual tactile spatial perception during a crossmodal exposure
Source: Front Neural Circuits. 2022 Nov 10;16:933455. doi: 10.3389/fncir.2022.933455 (PMC9684216; doi:10.3389/fncir.2022.933455)
Supplement: Supplementary file 1 [file Data_Sheet_1.docx]

**Appendix A**

**A.1 Mathematical Description**

Since the model presents two symmetrical networks, only the equations for one hemisphere (the left one) will be presented. The superscripts *t*, *v*, and *m* will identify elements referring to tactile, visual, and multisensory excitatory elements, respectively; the superscript *g* will indicate quantities referring to inhibitory interneurons; the superscripts *B* and *D* will distinguish the Bright-LED stimulated and Dim-LED stimulated hemisphere; the subscripts *ij* or *hk* will represent the spatial position of individual neurons.

Table A1 - Basal Parameter Values

| *Receptive Fields* | | | | | | | |
| --- | --- | --- | --- | --- | --- | --- | --- |
| A = 1 | | | | σ = 0.5cm | | | |
| *Tactile Input* | | | *Bright Visual Input* | | *Dim Visual Input* | | |
| I0_t_ = 6 | σ_t_ = 0.3 | | I0_B_ = 6 | σ_B_ = 0.3 | I0_D_ = 2.4 | | σ_D_ = 0.3 |
| *Lateral Synapses Unisensory Regions* | | | | | | | |
| L_ex_ = 0.15 | | σ_ex_ = 2 | | L_in_ = 0.05 | | σ_in_ = 8 | |
| *Excitatory Feedback Synapses* | | | | *Inhibitory Feedback Synapses* | | | |
| W = 0.5 | | | | I = 0.8 | | | |
| *Excitatory Feedforward Synapses* | | | | *Excitatory Inter-hemisphere Synapses* | | | |
| Wm = 0.8 | | | | Wi = 0.3 | | | |
| *Unisensory Neurons* | | | | | | | |
| fmin = -0.6 | | fmax = 5 | | s = 19.43 | | p = 0.34 | |
| *Multisensory Neurons* | | | | | | | |
| fmin = 0 | | fmax = 5 | | s = 12 | | p = 0.6 | |
| *Interneurons* | | | | | | | |
| fmin = 0 | | fmax = 5 | | s = 2.5 | | p = 1.32 | |
| *Temporal Dynamics* | | | | | | | |
| τ = 20ms | | | | Delay = 20ms | | | |

*Organization of the unisensory and multisensory regions*.

The unisensory areas are composed by NsxMs neurons (*s = t, v*), with Ns = 20, Ms = 40. In both areas, the neurons’ RFs differ from one another of 0.5 cm, along the x and y directions. Hence, the tactile and the visual areas cover a space of 10 cm by 20 cm, representing the surface of one hand and the corresponding visual space, in an extremely simplified form. In the following, we will denote with xi and yj the center of the RFs of a generic neuron *ij*. By considering a reference frame rigidly connected with the hand (see Figure A1), we can write:

x_ti_ = i·0.5cm (i=1, 2, ..., Ns); y_tj_ = j·0.5cm (j=1, 2, ..., Ms) *s = t, v*;

for the tactile and visual neurons.

Moreover, for simplicity, we assumed that the overall visual-tactile representation of the hand, coded in the multisensory region, is represented by just a single multisensory element (N_m_ = 1; M_m_ = 1), receiving excitation from every tactile and visual neural unit. Similarly, a single inhibitory interneuron is considered in each hemisphere, implementing the inter-hemisphere competition.


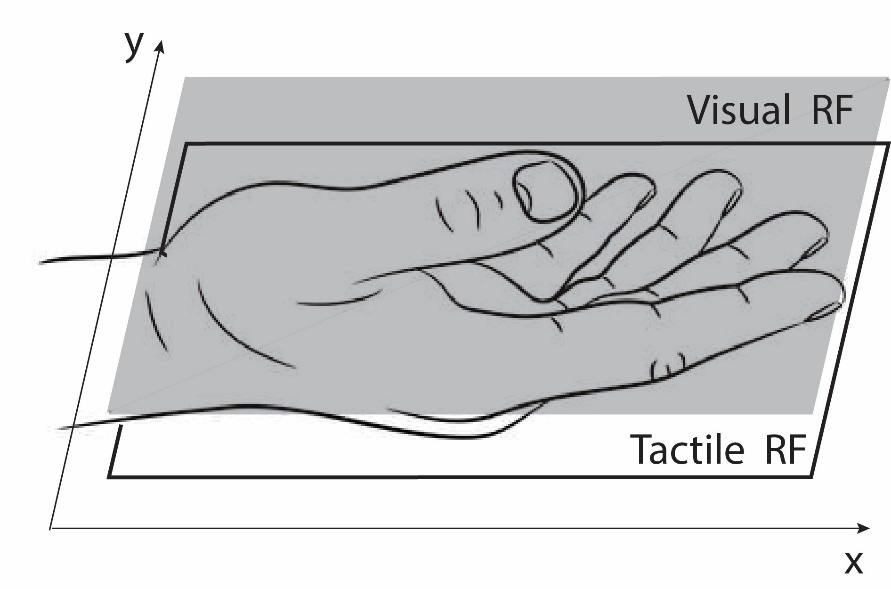


Figure A1 – Visual and Tactile RFs of a simulated hand. The two unisensory representations have the same dimensions along the x and y axes and cover the whole surface of the hand.

*Receptive fields of unisensory neurons*.

Hereinafter, each RF will be denoted with the symbol $\Phi$, and is described with a gaussian function. Hence, for a neuron *ij* in the generic unisensory area *s* (*s = t, v*) within the “Bright-LED” hemisphere, its RF is described by the following equation:

$\Phi_{ij}^{s, B}\left( i,j \right)= \Phi_{0}^{s, B}\cdot exp\left( -\frac{\left( x_{i}^{s,B}-x \right)^{2}+\left( y_{j}^{s,B}-y \right)^{2}}{2\cdot\left( \sigma_{\Phi}^{s,B} \right)^{2}} \right), s=t, v;$ (A.1)

Where *x_i_, y_j_* identify the center of the RF, *x* and *y* are the spatial coordinates, and $\Phi_{0}^{s, B}$and $\sigma_{\Phi}^{s,B}$ represent the amplitude and standard deviation of the gaussian function (three standard deviations approximately cover the overall RF). According to equation A.1, an external stimulus applied at the position *x, y* excites not only the neuron centered in that position but also the proximal neurons with RFs covering that portion of the external space.

*Unisensory neurons*.

A generic unisensory neuron *ij* in the Visual or Tactile area is stimulated by an overall input that is the sum of four elements:

- $\varphi_{ij}(t)$, the excitation produced by the external stimulus, that depends on the neuron’s RF $\Phi_{ij}$;
- $\lambda_{ij}(t)$), the effect of the lateral synapses linking elements in the same area;
- $\beta_{ij}(t)$, the excitatory component due to the feedback excitatory projections from the multisensory neuron;
- $\gamma_{ij}(t)$, the inhibition coming from the interneurons through their inhibitory feedback synapses.

Each contribution will be described below.

The input $\varphi_{ij}^{s,B}$ to the *ij*-neuron, caused by an external stimulus, is computed as the inner product of the stimulus and the corresponding neuron’s RF, according to the following equation:

$\varphi_{ij}^{s,B}\left( t \right)= \iint\Phi_{ij}^{s,B}\left( x,y \right)\cdot I^{s.B}\left( x,y,t \right)dxdy\cong\sum_{x} \sum_{y} \Phi_{ij}^{s,B}\left( x,y \right)\cdot I^{s.B}\left( x,y,t \right)\Delta x\Delta y, s=t, v;$ (A.2)

Where $I^{s,B}(x,y,t)$ is the external stimulus (tactile or visual) presented to the network, in position (x, y), at time t, and it is obtained by a two-dimensional gaussian function:

$I^{s,B}\left( x,y,t \right)= \left\{ \begin{aligned} 0, t<t_{0} \\ I_{0}^{s,B}\cdot\exp\left( -\frac{\left( x_{0}^{s,B}-x \right)^{2}+\left( y_{0}^{s,B}-y \right)^{2}}{2\cdot\left( \sigma_{I}^{s,B} \right)^{2}} \right), t\geq t_{0} \end{aligned} \right.$ (A.3)

Where *t_0_* is the instant of stimulus application, *x_0_, y_0_* is the central point of the stimulus, and $I_{0}^{s,B}$ and $\sigma_{I}^{s,B}$ represent its amplitude and standard deviation. We used a small standard deviation (see Table A1) to mimic a punctual external stimulus.

The input that a unisensory neuron receives from other neurons in the same area via the lateral synapses, $\lambda_{ij}(t)$), is defined as

$\lambda_{i,j}^{s,B}\left( t \right)=\sum_{h=1}^{N^{s}} \sum_{k=1}^{M^{s}} \Lambda_{ij,hk}^{s,B}\cdot z_{hk}^{s,B}\left( t \right), s=t,v$ (A.4)

where $z_{hk}^{s,B}\left( t \right)$represents the activity of the *hk*-neuron in the area *s* (*s = t, v*) of the Bright-LED hemisphere (computed below). $\Lambda_{ij,hk}^{s,B}$ indicates the strength of the connection between the presynaptic neuron, at the position *hk*, and the postsynaptic neuron at the position *ij*. These synapses are symmetrical and are arranged according to a Mexican hat function:

$\Lambda_{ij,hk}^{s,B}=\left\{ \begin{aligned} \Lambda_{ex}^{s,B}\cdot\exp\left( -\frac{\left( x_{i}^{s,B}-x_{h}^{s,B} \right)^{2}+\left( y_{j}^{s,B}-y_{k}^{s,B} \right)^{2}}{2\cdot\left( \sigma_{ex}^{s,B} \right)^{2}} \right)-\Lambda_{in}^{s,B}\cdot\exp\left( -\frac{\left( x_{i}^{s,B}-x_{h}^{s,B} \right)^{2}+\left( y_{j}^{s,B}-y_{k}^{s,B} \right)^{2}}{2\cdot\left( \sigma_{in}^{s,B} \right)^{2}} \right), ij\neq hk \\ 0, ij=hk \end{aligned} \right.$ (A.5)

Parameters $\Lambda_{ex}^{s,B}$ and $\sigma_{ex}^{s,B}$ (*s = t, v*) define the excitatory gaussian function and parameters $\Lambda_{in}^{s,B}$ and $\sigma_{in}^{s,B}$ (*s = t, v*) the inhibitory one: they establish the strength and extension of these synapses. The null term in equation 2.5 avoids autoexcitation.

The effect of the feedback excitatory synapses from the multisensory area to the unisensory neurons, $\beta_{ij}(t)$, is computed as:

$\beta_{ij}^{s,B}\left( t \right)=B_{ij}^{s,B}\cdot z^{m,B}\left( t \right), s=t,v$ (A.6)

$z^{m,B}\left( t \right)$ represents the activity of the *hk* neuron in the multisensory area (computed below). $B_{ij}^{s,B}$ indicates the strength of the synaptic connection from the presynaptic multisensory neuron to the postsynaptic *ij*-neuron, in the unisensory area *s* (s = t, v). For simplicity, in this work we assume that the synaptic connections between the unisensory and multisensory regions present a uniform distribution; that is, the synaptic strength is equal for every postsynaptic neural element. Accordingly, we have:

$B_{ij}^{s,B}=B_{0}^{s,B}, s=t,v$ (A.7)

Where $B_{0}^{s,B}$ set the effectiveness of the synapses.

Finally, unisensory input neurons receive a feedback input from an inhibitory interneuron,

$\gamma_{i,j}^{s,B}\left( t \right)=\Gamma_{ij}^{s,B}\cdot z^{g,B}\left( t \right), s=t,v$ (A.8)

Where $z^{g,B}\left( t \right)$represents the activity of the inhibitory interneuron. This activity depends on the visual-tactile information at the other hemisphere (see below). $\Gamma_{ij}^{s,B}$ is the strength of the synaptic connection from the interneuron to neuron *ij* in the unisensory area *s* (tactile or visual). We assume that this inhibition equally affects every unisensory neuron in the tactile and visual input regions. Accordingly:

$\Gamma_{ij,hk}^{s,B}=\Gamma_{0}^{s,B}, s=t,v$ (A.9)

The overall input, $u_{ij}^{s}(t)$, received by a unisensory neuron, is the sum of the four previous terms:

$u_{ij}^{s,B}\left( t \right)=\varphi_{ij}^{s,B}\left( t \right)+\lambda_{i,j}^{s,B}\left( t \right)+\beta_{ij}^{s,B}\left( t \right)+\gamma_{i,j}^{s,B}\left( t \right)$ (A.10)

From this input, the neuron’s activity is computed through a first-order dynamics and a static sigmoidal relationship:

$\tau\frac{dq_{ij}^{s,B}(t)}{dt}=-q_{ij}^{s,B}\left( t \right)+u_{ij}^{s,B}(t), s=t,v$ (A.11)

$z_{ij}^{s,B}\left( t \right)= \frac{f_{min}^{s}+f_{max}^{s}\cdot e^{\left( \left( q_{ij}^{s,B}-Q^{s} \right)\cdot r^{s} \right)}}{\left( 1+e^{\left( \left( q_{ij}^{s,B}-Q^{s} \right)\cdot r^{s} \right)} \right)}\cdot H\left( \frac{f_{min}^{s}+f_{max}^{s}\cdot e^{\left( \left( q_{ij}^{s,B}-Q^{s} \right)\cdot r^{s} \right)}}{\left( 1+e^{\left( \left( q_{ij}^{s,B}-Q^{s} \right)\cdot r^{s} \right)} \right)} \right), s=t,v$ (A.12)

Where *q* represents the state variable. Parameters $f_{min}^{s}$ and $f_{max}^{s}$ set the lower and upper saturation of the sigmoidal function, $r^{s}$ and $Q^{s}$ are the slope and the value of the input at the central point of the sigmoid, respectively. Since parameter $f_{min}^{s}$ has a negative value (see Table A1), the Heaviside function H( ) has been introduced to avoid neuron activity from becoming negative. Hence, the activity of a unimodal neuron is equal to zero until its total input overcomes a given threshold. Parameter τ in equation A.11 is the time constant of the differential equation.

*The multisensory neuron*

This neural element receives inputs from every neuron of the two unisensory areas belonging to the same hemisphere, via feedforward synapses. Its overall input is computed as:

$u^{m,B}\left( t \right)=\sum_{i=1}^{Nt} \sum_{j=1}^{Mt} W_{ij}^{t,B}\cdot z_{ij}^{t,B}\left( t \right)+\sum_{i=1}^{Nv} \sum_{j=1}^{Mv} W_{ij}^{v,B}\cdot z_{ij}^{v,B}\left( t \right)$ (A.13)

$z_{ij}^{s,B}\left( t \right)$ (*s = t, v*) represents the activity of the neuron ij in the unimodal (tactile or visual) area, computed through equations A.11 and A.12. $W_{ij}^{s,B}$ denotes the feedforward excitatory synapses from the unisensory neuron to the multimodal neuron. For simplicity, these synapses are described by an arrangement, similar to those implemented for the excitatory and inhibitory feedback connections to the unisensory elements:

$W_{ij}^{s,B}=W_{0}^{s,B}, s=t,v$ (A.14)

Where $W_{0}^{s,B}$ set the effectiveness of the synapses targeting the multisensory element, and it has been chosen equal for the visual and tactile pathway.

The activity of a multisensory neuron is computed from its input by using equations like A.11 and A.12:

$\tau\frac{dq^{m,B}(t)}{dt}=-q^{m,B}(t)+u^{m,B}(t),$ (A.15)

$z^{m,B}(t)= \frac{f_{min}^{m}+f_{max}^{m}\cdot e^{\left( \left( q^{m,B}-Q^{m} \right)\cdot r^{m} \right)}}{\left( 1+e^{\left( \left( q^{m,B}-Q^{m} \right)\cdot r^{m} \right)} \right)}$ (A.16)

The meaning of the symbols is the same as in equations A.11 and A.12.

*The inhibitory interneuron.*

The inhibitory interneuron in one hemisphere receives synapses from the multisensory neuron in the other hemisphere. Hence, the input to the interneuron in the Bright-LED hemisphere is

$u^{g,B}\left( t \right)=X^{D}\cdot z^{m,D}(t-d)$ (A.17)

Where $z^{m,D}(t)$ is the activity of the multisensory neuron in the Dim-LED hemisphere and *d* is a pure delay, simulating the time necessary for projections to cross the inter-hemispheric structures and become effective. $X^{D}$ represents the strength of the cross-connection linking the multimodal neuron in the Dim-LED hemisphere to the inhibitory interneuron in the Bright-LED hemisphere.

Then, equations like Eqs. A.11 and A.12 are used to compute the activity of the interneuron:

$\tau\frac{dq^{g,B}(t)}{dt}=-q^{g,B}(t)+u^{g,B}(t),$ (A.18)

$z^{g,B}(t)= \frac{f_{min}^{g}+f_{max}^{g}\cdot e^{\left( \left( q^{g,B}-Q^{g} \right)\cdot r^{g} \right)}}{\left( 1+e^{\left( \left( q^{g,B}-Q^{g} \right)\cdot r^{g} \right)} \right)}$ (A.19)

The meaning of the symbols is the same as in equations A.11 and A.12.
